# Supplementary material for: A gene‐centered C. elegans protein–DNA interaction network provides a framework for functional predictions
Source: Mol Syst Biol. 2016 Oct 26;12(10):884. doi: 10.15252/msb.20167131 (PMC5081483; doi:10.15252/msb.20167131)
Supplement: Supplementary file 1 — Expanded View Figures PDF [file MSB-12-884-s001.pdf]

Expanded View Figures

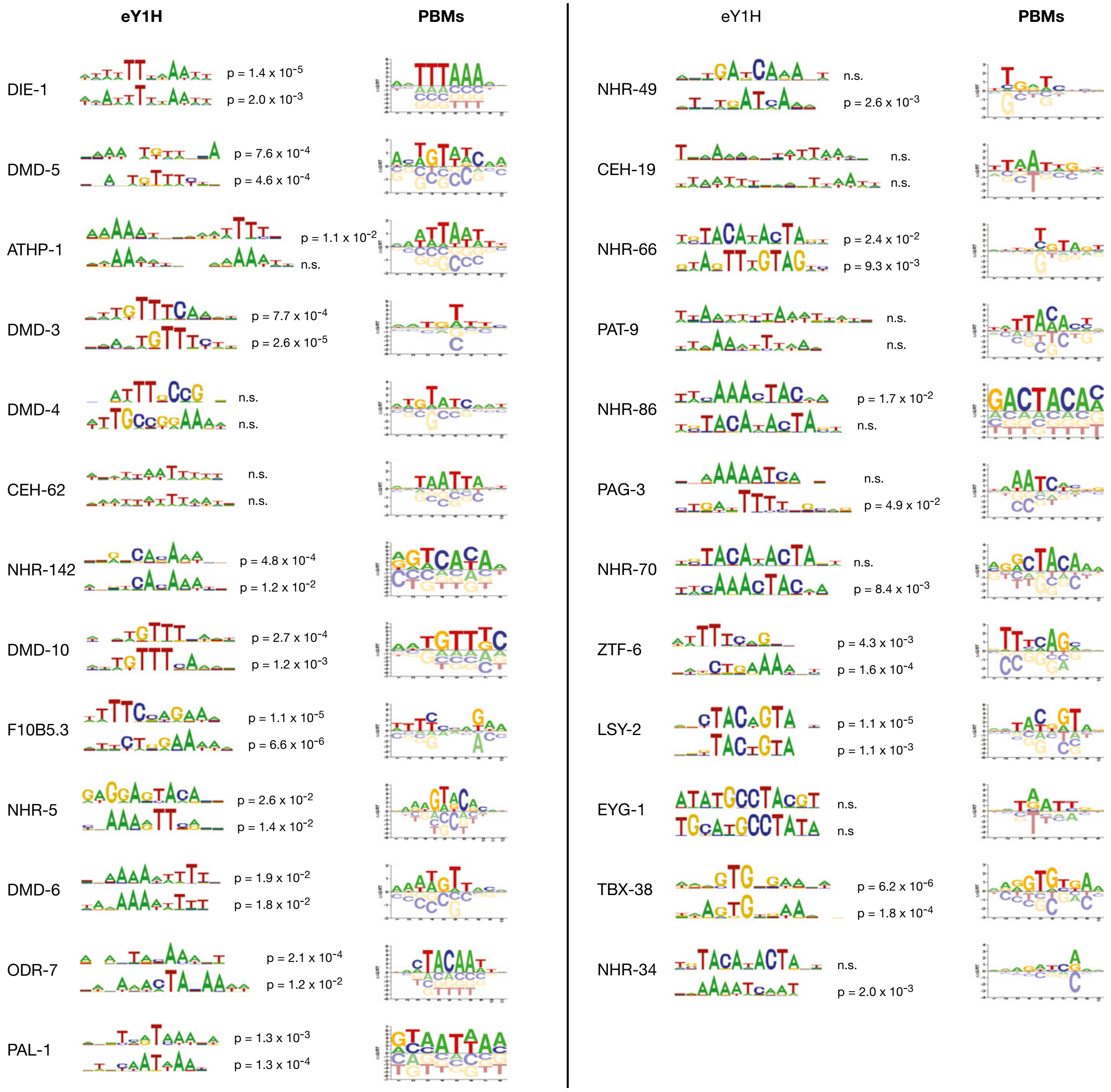

Figure EV1. Comparison between motifs derived from eY1H and PBM data.
For each TF, the two top-scoring motifs derived from eY1H data are compared to the motif logos derived from PBM data reported in CisBP (Weirauch *et al*, 2014). Motifs are shown for all the TFs with more than 50 eY1H targets and with available motifs derived from PBM data. Statistical significance between the eY1H- and PBM-derived motifs was determined using the TOMTOM software version 4.11.2.

 © 2016 The Authors

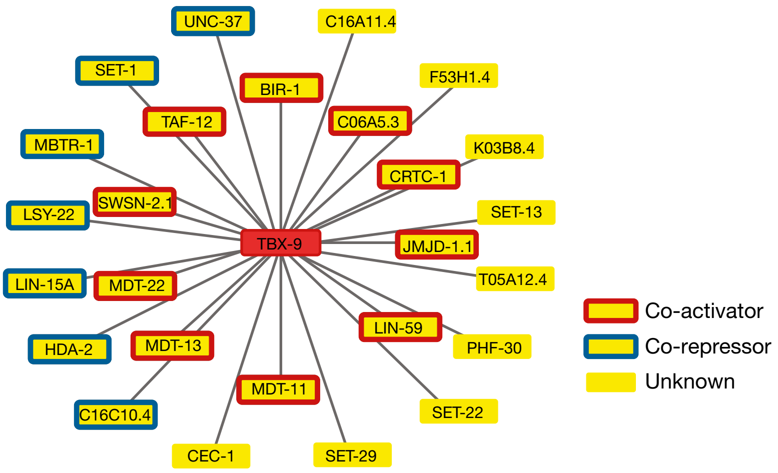

Figure EV2. Cofactor interactions with TBX-9.
Protein–protein interactions between the TF TBX-9 and cofactors from Reece-Hoyes *et al* (2013).

Figure EV3. TF association network.
Each node represents a TF, and edges connect TFs with a target profile similarity ≥ 0.2. TFs with degree ≥ 3 in the eY1H network are shown. Node color indicates TF families. Essential TFs are highlighted by a black outline. bHLH, basic helix-loop-helix; bZIP, basic leucine zipper domain; HD, homeodomain; NHR, nuclear hormone receptor; PD, paired domain; WH-ETS, winged helix E26 transformation-specific; ZF-C2H2, zinc finger C2H2.

© 2016 The Authors

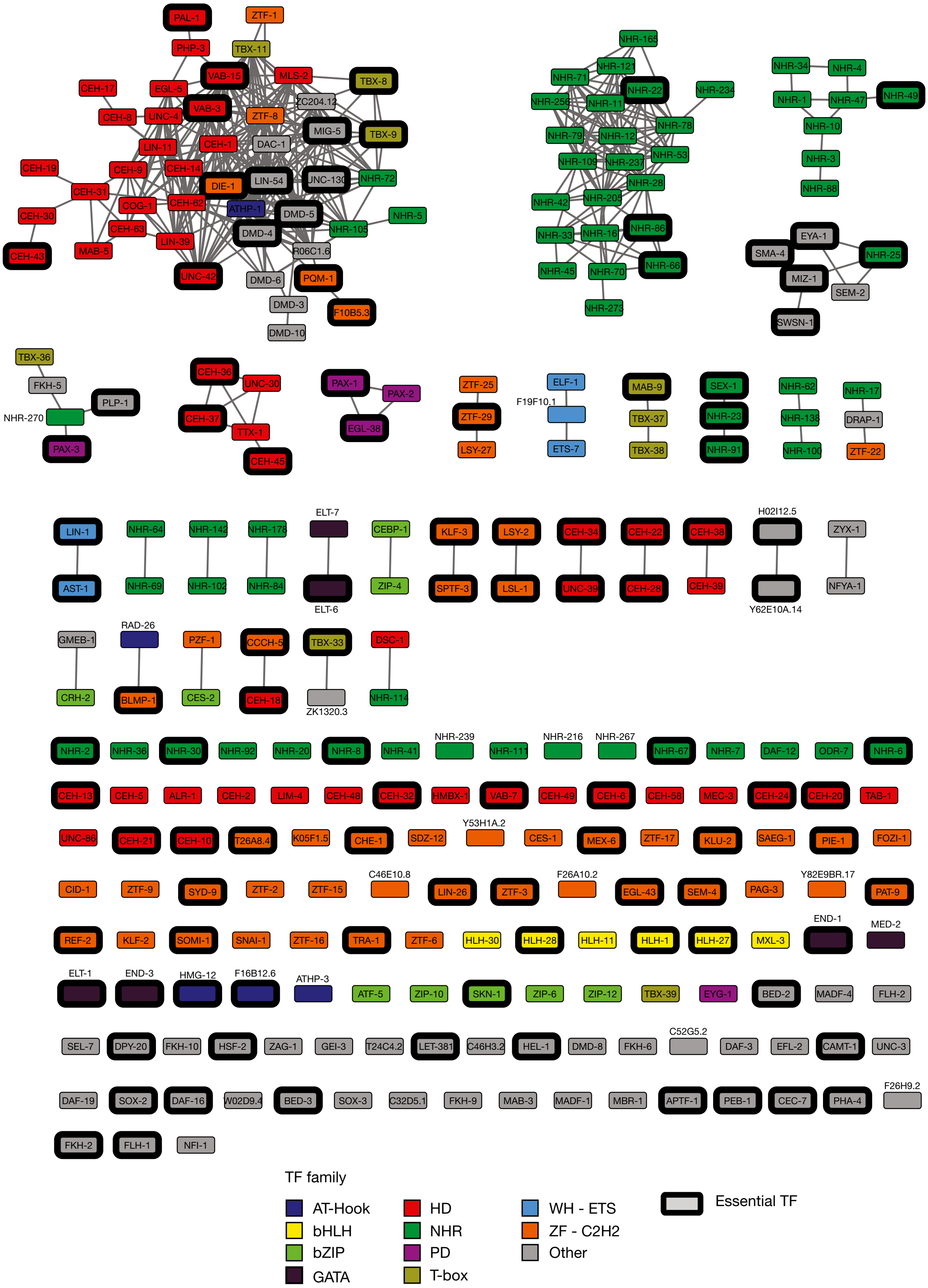

Figure EV3.

 © 2016 The Authors
